# Payment options: An analysis of 6 years of payment plan data and potential implications for for-profit clinics, non-profit veterinary providers, and funders to access to care initiatives

**DOI:** 10.3389/fvets.2022.895532

**Published:** 2022-08-01

**Authors:** Heather J. Cammisa, Samantha Hill

**Affiliations:** ^1^Open Door Veterinary Collective, Grand Rapids, OH, United States; ^2^Independent Researcher, Bedford, OH, United States

**Keywords:** access to care, credit, payment options, funder, financing, non-profit

## Abstract

Analyzing a dataset of payment plans disassociated with traditional credit scoring, this research, for the first time, offers insights into the mitigation of cash flow and credit ineligibility challenges in access to veterinary care. Specifically, this paper explores financial fragility among pet families and whether payment options offer substantial bridges in access to care challenges for veterinarians and clients. Researchers introduce a veterinary care multiplier to estimate the potential increase in veterinary care that may be provided by for-profit and non-profit clinics from additional payment options. The implications for non-profits working to address access to care is that by directing donor dollars to cover the 6.9% that is potentially left unpaid in meeting pet families simply facing cash flow challenges, a non-profit clinic could provide 14.5 times the veterinary care vs. full subsidies. In *for-profit* clinics, allocating some of a clinic's discount budget may similarly yield 14.5 times the care for clients likely to be declined by the traditional credit options. Further research is recommended to explore how deeply these options penetrate all financially fragile pet owners and outcomes in the absence of these tools for credit-declined clients. Additional research to determine the levels at which payment options reduce economic euthanasia decisions, reduce the client and staff stress, increase the value perception and compliance with suggested care, enable better outcomes for patients, and increase clinic revenue is also recommended. The researchers conclude that payment options that are independent of traditional credit scoring mitigate financial barriers to obtaining veterinary care.

## Introduction

According to the American Pet Products Association (1), pet ownership continues to increase with 70%, or 90.5 million households in the United States owning a pet in 2021–2022. This is up from 56% in 1986. Access to care research has found that one in four pet families (27.9%) were unable to access needed veterinary care in the previous 2 years (2). Increasingly, research is identifying the inability to access care among select populations, such as homebound seniors receiving food assistance, found to be nearly one in two clients with pets (44.8%) (3). The primary barrier for preventative, sick, and emergency care across age groups, geography, and other population views is consistently financial. The cost of services is not the sole driver of this top barrier. This research discusses the cash flow crisis of care and the inability to access traditional credit options to cover the cost of care as a mitigable barrier by offering insights, for the first time, from an in-depth analysis of empirical client payment plan data. Specifically, this paper explores whether payment options offer substantial bridges in access to care challenges for veterinarians and clients and estimates the potential increase in veterinary care that may be provided by for-profit and non-profit providers from additional payment options to address financial fragility among pet families who may not qualify for traditional credit.

Cash flow and available funds crises among American consumers are found to be common and present across income levels. The term “financially fragile” is used to describe those who could not come up with $2,000 in 30 days (4). In a 2018 study from George Washington University (5), approximately 36% of working adults are found to be financially fragile. A recent national study finds that this is reflected among pet owners at all income levels with one-third of those surveyed reporting that they were “not at all confident” or “only slightly confident” they could come up with $2,000 if an unexpected need arose (6). This study found that financial fragility was a significant predictor of perception of ease in accessing veterinary care and was found at all household income levels. Another often-quoted statistic is the number of American workers living paycheck to paycheck. In a December 2021 survey, six in ten American consumers were found to live paycheck to paycheck. Even among those earning in excess of $100,000, approximately 42% were found to be living paycheck to paycheck. The largest group found living paycheck to paycheck were millennials, at 70% (7). Millennials comprise the largest age segment of pet ownership at 32% according to the American Pet Products Association (1). Financial fragility is widespread among the public and pet owners and, thereby presenting a major barrier to veterinary care.

The common “solutions” in financing veterinary care are inadequate for pet owners with cash flow challenges and an inability to qualify for traditional credit programs. In lieu of personal savings or credit cards, the primary options for pet owners to pay for veterinary services are pet insurance, discounts, or credit-based payment programs. Pet insurance, with premiums paid monthly or annually, most commonly requires owners to pay out of pocket and later be reimbursed for expenses rather than paying the clinic directly as in human healthcare. Discounts may be offered, but owners face the remaining cost of services for immediate payment. Third-party credit (TPC) programs, which pay the clinics directly (minus a percentage as a service fee), are available for owners who qualify and these owners may face interest rates of 26.9%. In a recent study by Bir et al. (8) owners preferred discounts to these TPC programs when considering hypothetical costs of routine and non-routine care, but this study did not consider practice-led lending (PLL) programs, which are not credit-based.

In monetary economics, a money multiplier identifies the potential expansion of the money supply in the economy based upon a reserve amount, the “reserve ratio,” banking institutions must hold on hand to cover deposit accounts (9) https://www.investopedia.com/terms/r/reserveratio.asp. When the reserve ratio is lowered, money is “created” by banks being able to lend more dollars and, thereby increasing the money supply. Similarly, when payment options are offered in veterinary practices that bridge the gap in consumer cash flow and financial fragility, we may look at the default ratio to assess what expansion of care is possible when that default rate (a reserve rate) is covered, either by risk tolerance, donor dollars or funds that are otherwise earmarked in practice budgets for discounts to clients.

As the default amount decreases, a practice is able to expand its offering of care to clients that need payment options to access care. At its maximum, non-credit-based payment options for those who would not qualify for traditional credit and where other funds are not obtainable, the care multiplier is (1/default rate). More research is needed to identify the experienced decline rate of TPC and what alternatives clients face in the absence of payment options when declined by TPC to understand the final impact PLLs may have in covering the gap in accessing care due to finances. The expansion of fund potential is illustrated in the following examples:

Example A: A practice budgets $10,000 for discounts per year. If, instead, the practice used those funds to cover potential defaults on PLLs for clients declined by TPCs and the default rate on PLL tools was found to be 5%, then the practice would have $200,000 of care that it could provide and would receive a net of $190,000 in payments from clients.

$10,000 budgeted discount dollars ^*^ (1/0.05) multiplier = $200,000 of potential care.
$200,000 of potential care – $10,000 payments defaulted = $190,000 net revenue.

Example B: A non-profit provider has received a $75,000 grant to provide veterinary care to low-income residents of a city. The provider could subsidize the entire cost of care and provide $75,000 of care to clients at no cost. If, instead, the provider provided PLL options such as “buy now, pay later” payment plans and used the donor's dollars to cover a 15% default rate, the provider could instead provide $500,000 of veterinary care to low-income pet families in the city. A $425,000 increase.

$75,000 donor dollars ^*^ (1/0.15) multiplier = $500,000 of potential care.
$500,000 of potential care – $75,000 donor dollars = $425,000 increase over full subsidy of care.

Credit scoring tiers are identified by the Bureau of Consumer Finance Protection (10) as Superprime (720 +), Prime (660–719), Near-prime (620–659), Sub-prime (580–619), and Deep subprime (<579). Not all adults are scorable with the term “credit invisible” referring to those who have no credit history and “unscorable” referring to those who have stale or “too thin” credit histories to be scored. Traditional credit financing looks for credit scores at or above Near-prime (620+). According to a 2019 Bureau of Consumer Financial Protection report, four in ten US adults have credit scores under 620 or are unscorable or credit invisible. This signals that only 60% of the US adults may qualify for traditional financing options in use in veterinary practices that are dependent on the credit scores.

This research examines 6 years of unscored and soft-credit-scored payment plan account data across veterinary clinic types, analyzing payment plan usage and default rates and, using the care multiplier, providing estimations of potential impacts on clinics, clients, and access to veterinary care initiatives.

### Methodology

We utilize the following descriptions to differentiate between traditional credit-based financing and a practice-based financing option:

Practice-led lending—where the practice extends credit and may use an outside management service to obtain lending recommendations, with or without a soft credit check, and to manage client billing. The practice is not charged a fee on the financed amount but does face the risk of defaults.

Third-party credit—where an outside company offers credit using its own creditworthiness testing, based on credit scores. The practice is charged a fee, typically 5–10% of the financed amount, and does not face the risk of defaults.

A dataset from a PLL option, VetBilling, of 21,225 unique veterinary client accounts with first-payment dates between 2016 and 2021 was analyzed in this research. These accounts are distributed among 397 unique veterinary provider organizations and are scrubbed of personal identification information.

Accounts have statuses of Active (currently open) or Closed (no longer active). Closed accounts have sub-statuses based on the standing of the account: Expired/Canceled (account was paid in full); Collect (account was sent to the collection after 90 days of unpaid balance); Write-Off (WO) (account written off by the client organization). An additional indicator for Expired/Canceled accounts is provided for when the account was paid in full after being sent to collection.

Account data analyzed in the dataset include Total Cost of Services (total amount of the services provided), Down Payment Amounts (amount account holder paid up front), Total Financed Amounts (amount included in the payment plan, i.e., total cost of services minus down payment amount), Term Length (number of months), Financed Amount Remaining (portion of the total financed amount that is remaining as of December 2021, i.e., total financed amount minus total amount of payments made), Financed Amount Past Due (total amount not paid by due date), WO Amount (total amount not paid on the account), and Credit Score Recommendation (soft credit check). Default amounts for accounts in Collect were calculated using an aggregate of Financed Amount Remaining and Financed Amount Past Due. WO Amounts were used for accounts in WO status for default amounts.

Credit Score Recommendations (CSR) codes are optional credit ratings for clinics to run on an account. The rating uses a soft credit check along with VetBilling's internal scoring system that considers valid phone number, address, email, banking information, and payment history with financing companies, if any. The score also takes into account how the contract was signed (manually or *via* phone ID). Scores assigned are A+, A, B, C, D, E, F, and G. While it is not possible to directly map the eligibility for traditional credit-based financing or credit scores since companies keep this proprietary information confidential; VetBilling has advised that E, F, and G ratings would be very likely rejected by credit-based lenders and D ratings are somewhat likely as well. Those with A+, A, B, and C may be eligible for other credit-based financings.

Client organizations self-selected their organization type: small animal clinics, emergency services clinics, non-profit clinics, and other clinics (specialty, mobile, or mixed-animal type clinics).

The analysis of payment default rates looked only at accounts with a status of Closed, whereby the ultimate outcome is known.

Data were analyzed for summary statistics using Python.

## Results

### General

The 397 unique client organizations broke down as follows: 332 (83.6%) were small animal clinics, 39 (9.8%) were emergency services clinics, 14 (3.5%) were non-profit clinics, and 12 (3.0%) were other clinics.

The 21,225 accounts in the dataset broke down as 2,846 (13.4%) were Active and 18,379 (86.6%) were Closed. Of the Closed accounts, 16,901 (92.0%) were Expired/Canceled, 1,249 (8.0%) were listed as Collect/WO. Of the accounts that were Expired/Canceled, 2.3% of these accounts were originally sent to collection and then paid in full. Active accounts total is $3,634,777 in financed amounts, and closed accounts total is $13,026,943 in financed amounts.

### Organization type profiles

Over the 6-year period, small animal veterinary clinics have been the primary clients utilizing the PLL option. While small animal clinics make up the majority of accounts each year (79.6% in 2016 and 55.7% in 2021), there has been an increase in accounts held by non-profit clinics (from 2.5% in 2016 to 15.9% in 2021), and emergency clinics (from 17.3% in 2016 to 27.5% in 2021).

Of the three main organization types, emergency clinics had the highest percentage of total accounts that are currently active (20.5%). Emergency clinics have higher financed amounts than the other organization types. The average financed amount for total accounts (active and closed) at emergency clinics is $1,107, which is 44.7% more than small animal clinics ($765) and 172.7% more than non-profit clinics ($406).

As a percentage of the total cost of services, non-profit clinics finance a lower portion, on average, than other organization types. Of the total accounts, the average financed percentage of the total costs was 53.1% for non-profit clinics, 75.9% for small animal clinics, and 68.5% for emergency clinics.

While emergency clinics have a higher down payment amount overall, which they determine with or without a recommendation from the PLL manager, non-profit clinics have a higher average percentage of total cost covered by the down payment than any other organization type. Non-profit clinics have an average of 50% as a down payment for services. Payment plans at emergency clinics and small animal clinics have an average of 35.6 and 24.9%, respectively, as a down payment.

Non-profit clinics average is 5.3 months payment terms from total accounts, while small animal clinics average is 7.8 months and emergency clinics average is 8.0 months. Even though emergency clinic accounts have average higher financed amounts than small animal clinics, their payment term average is very similar. (Table 1: Organization type profile). Further information is provided in the Supplementary Materials.

**Table 1:** Organization type profiles.

**Org type**	**Account status (as of 12/27/2021)**	**# accounts**	**Percentage of org type accounts (%)**	**Average term**	**Median term**	**Total $ financed**	**Percentage of Org type total financed amount (%)**	**Average financed amounts**	**Median financed amount**	**Average down payment**	**Median down payment**	**Down payment % of total cost**
Emergency services (*n* = 39)	Total	4,498		8.0	6	$4,979,129		$1,107	$679	$613	$255	35.6
	Active accounts	924	20.5	12.0	12	$1,472,638	29.6%	$1,594	$1,194	$684	$329	30.0
	Closed accounts	3,574	79.5	6.9	6	$3,506,491	70.4%	$981	$577	$595	$250	37.7
	*Canceled/expired*	*3,178*	*88.9*	6.7	6	*$2,989,332*	*85.3%*	$941	$555	$609	$250	39.3
	*Collect/WO*	*396*	*11.1*	8.7	6	*$517,159*	*14.8%*	$1,306	$805	$479	$175	26.8
Non-profit (*n* = 14)	Total	3,262		5.3	4	$1,325,029		$406	$265	$405	$240	50.0
	Active	339	10.4	9.2	6	$201,368	15.2%	$594	$415	$467	$229	44.0
	Closed	2,923	84.8	4.8	4	$1,123,661	89.6%	$384	$256	$398	$240	50.9
	*Canceled/Expired*	*2,747*	*94.0*	4.7	4	*$1,034,771*	*92.1%*	377	$252	393.9	$240	51.1
	*Collect/WO*	*176*	*6.0*	5.9	5	*$88,891*	*7.9%*	$505	$302	$467	$289	48.0
Small animal (*n* = 332)	Total	13,217		7.8	6	$10,106,670		$765	$509	$254	$120	24.9
	Active	1,560	11.8	13.9	11	$1,941,657	19.2	$1,245	$861	$265	$75	17.5
	Closed	11,657	88.2	7.0	6	$8,165,014	80.8	$700	$480	$252	$126	26.5
	*Canceled/Expired*	*10,756*	*92.3*	6.8	6	*$7,216,356*	*88.4*	$671	$477	$255	$130	27.6
	*Collect/WO*	*901*	*7.7*	10.0	7	*$948,657*	*11.6*	$1,053	$675	$215	$50	17.0
Other (*n* = 12)	Total	248		9.1	6	$250,892		$1,012	$738	$335	$148	24.9
	Active	23	9.3	11.8	11	$19,115	7.6	$831	$929	$207	$112	19.9
	Closed	225	90.7	8.8	6	$231,778	92.4	$1,030	$728	$348	$150	25.2
	*Canceled/Expired*	*220*	*97.8*	8.7	6	*$221,760*	*95.7*	$1,008	$696	$345	$148	25.5
	*Collect/WO*	*5*	*2.2*	10.6	10	*$10,018*	*4.3*	$2,004	$1,736	$446	$300	18.2
Total	Total	21,225		7.5	6	$16,661,720		$785	$490	$354	$170	31.1
	Active	2,846	13.4	12.7	12	$3,634,777	21.8	$1,277	$897	$425	$125	24.9
	Closed	18,379	86.6	6.7	6	$13,026,943	78.2	$709	$448	$343	$172	32.6
	*Canceled/Expired*	*16,901*	*92.0*	6.5	5	*$11,462,219*	*88.0*	$678	$435	$346	$175	33.8
	*Collect/WO*	*1,478*	*8.0*	9.2	6	*$1,564,724*	*12.0*	$1,061	$677	$318	$117	23.0

### Distribution of accounts with credit ratings

Less than 15% of all accounts have a CSR rating that was run by the clinic when determining whether to grant the payment plan or how to structure it. Specifically, there are 2,931 accounts (13.8% of all accounts in the dataset) that have a CSR score of A+, A, B, C, D, E, F, or G. Of the scored accounts, 87.4% (2,561) are closed.

Of the total accounts that were assigned a CSR score (active and closed), 70.0% are D–G. There is insufficient information to thoroughly compare if rated accounts are representative of the entire dataset. The breakdown of accounts by clinic type is similar, with 59.3% of accounts having a CSR score through small animal clinics while 39.2% are through emergency clinics. We do see that as the rating decreases, the portion of accounts and financed amounts shift to be heavily emergency instead of small animal clinics as seen through the distribution of accounts by the CSR score (Table 2: Accounts with CSR by organization type).

**Table 2:** Accounts with Credit Score Recommendations (CSR) by organization type.

	**Percentage of total accounts (with CSR scores) by org type (%)**				
	**Emergency (*****n*** = **1,148)**	**Non-profit (*****n*** = **32)**	**Small animal (*****n*** = **1,738)**	**Other (*****n*** = **13)**	**Grand total (*****n*** = **2,931)**
**A+ (*n* = 29)**	**0.6**	**0.0**	**1.5**	**0.0**	**1.0**
A (*n* = 126)	1.9	10.3	8.1	0.0	4.9
B (*n* = 245)	8.0	0.0	11.5	27.6	9.7
C (*n* = 480)	14.6	12.3	21.3	23.1	17.8
D (*n* = 670)	22.1	17.6	21.6	15.5	21.8
E (*n* = 613)	23.8	4.6	17.3	11.0	20.6
F (*n* = 374)	14.9	28.8	8.8	15.5	12.1
G (*n* = 394)	14.1	26.4	10.1	7.3	12.2
	100.0	100.0	100.0	100.0	100.0

### Default accounts and amounts

As of December 2021, 92.0% of all closed accounts were paid in full, leaving 8.0% that ultimately went to default. In terms of the total cost of services provided, 94.0% was paid through down payments and monthly installments. The total amount defaulted represents 8.9% of total financed amounts and 6.0% of total cost of services. Non-profit clinics have a default rate of 2.8% of total cost of services for all closed accounts (Table 3: Default by organization type).

**Table 3:** Default by organization type.

	**Accounts in collection**			**Accounts in write-off (with write-off amounts)**			**All accounts**		
	**$ default**	**Percentage of total financed amount for accounts in Collection (%)**	**Percentage of total closed accounts financed amounts (%)**	**$ default**	**Percentage of total financed amount for accounts in WO (%)**	**Percentage of total closed accounts financed amounts (%)**	**Total cost of services (all closed accounts)**	**Total default**	**Default % of total cost of services**
Emergency	$304,008	74.9	8.7	$87,966	81.9	2.5	$5,632,077	$391,974	7.0
Non-profit	$60,194	74.0	5.4	$3,857	50.9	0.3	$2,287,926	$64,051	2.8
Small Animal	$587,249	74.7	7.2	$106,678	76.6	1.3	$11,106,160	$693,927	6.2
Other	$1,922	53.2	0.8	$4,662	72.8	2.0	$310,017	$6,584	2.1
Total	$953,473	74.7	7.3	$203,162	77.9	1.6	$19,336,181	$1,156,635	6.0

Accounts assigned a CSR score have a default rate of 4.9% while accounts without a CSR score have a default rate of 6.2%. The majority of closed accounts with a CSR score (70.2%) have a D, E, F, or G score, indicating that they are more likely to have been declined by TPC payment options. As CSR scores decrease, the default rate increases; however, as a group, D, E, F, and G accounts have a default rate of 6.9% of total cost of services (Table 4: Default by CSR Score).

**Table 4:** Default by CSR score.

	**Total Down payment**	**Total financed**	**Total cost of services**	**Total amount default**	**Downpayment % of total cost of services**	**Financed amounts % of total cost**	**Amount default % of financed amount**	**Amount default % of total cost**	**Percentage of total financed (CSR scores only) (%)**	**Percentage of total cost of services (CSR scores only) (%)**
A+ (*n* = 27)	$9,033.87	$25,466.24	$34,500.11	$0.00	26.2%	73.8	0.0	0.0	1.2	1.1
A (*n* = 116)	$48,232.92	$115,743.50	$163,76.40	$0.00	29.4%	70.6	0.0	0.0	5.4	5.2
B (*n* = 199)	$108,182.97	$193,216.20	$301,399.20	$1,883.59	35.9	64.1	1.0	0.6	9.0	9.5
C (*n* = 421)	$199,693.28	$382,107.50	$581,800.80	$8,829.01	34.3	65.7	2.3	1.5	17.8	18.3
D (*n* = 590)	$244,250.45	$461,680.50	$705,931.00	$28,357.54	34.6	65.4	6.1	4.0	21.5	22.2
E (*n* = 534)	$231,754.67	$446,115.40	$677,870.00	$40,531.63	34.2	65.8	9.1	6.0	20.8	21.3
F (*n* = 316)	$92,436.20	$230,434.20	$322,870.40	$25,839.60	28.6	71.4	11.2	8.0	10.7	10.1
G (*n* = 358)	$104,210.65	$289,687.30	$393,898.00	$50,981.60	26.5	73.5	17.6	12.9	13.5	12.4
A+,A,B,C (*n* = 763)	$365,143.04	$716,533.44	$1,081,676.51	$10,712.60	33.8	66.2	1.5	1.0	33.4	34.0
DEF (*n* = 1,440)	$568,441.32	$1,138,230.10	$1,706,671.40	$94,728.77	33.3	66.7	8.3	5.6	53.1	53.6
DEFG (*n* = 1,798)	$672,651.97	$1,427,917.40	$2,100,569.40	$1,45,710.37	32.0	68.0	10.2	6.9	66.6	66.0
EF (*n* = 850)	$324,190.87	$6,76,549.60	$1,000,740.40	$66,371.23	32.4	67.6	9.8	6.6	31.5	31.4
EFG (*n* = 1,208)	$428,401.52	$966,236.90	$1,94638.40	$1,17,352.83	30.7	69.3	12.1	8.4	45.1	43.8
CSR Score Total (*n* = 2,561)	$1,037,795.01	$2,144,451.00	$3,182,246.00	$1,56,423.00	32.6	67.4	7.3	4.9		
No CSR Score (*n* = 15,818)	$5,271,442.56	$1,0,882,492.00	$16,153,935.00	$1,000,213.00	32.6	67.4	9.2	6.2		
Total Dataset (closed accounts)	$6,309,237.57	$13,026,943.00	$193,36,181.00	$1,156,636.00	32.6	67.4	8.9	6.0		

## Discussion

The analysis offers insight into the expansion of veterinary care possible for both clients and clinics through the use of a PLL disassociated from traditional credit decision processes and where the client may make installment payments to mitigate the cash flow crises experienced by a large portion of the US adults.

Default rates varied by provider type and CSR; however, 91.1% of financed amounts were ultimately repaid, with 94.0% of total care costs paid *via* installments and down payments.

From an analysis of 2,561 accounts where CSR data are available, we can look to permutations of scores D–G (*n* = 1,798) to identify the potential care multiplier solely among pet families who most likely could not have qualified for TPC options.

Analyzing likely credit ineligibles at D, E, F, and G or the most conservative view that clients rated E, F, or G are likely to be ineligible for TPC options, the default amount as a ratio of total care provided is 6.9 and 8.4%, respectively, and the care multiplier is 14.5, or at minimum, 11.9 times the care that could otherwise be provided dollar for dollar by clinics and to pet families who may have had no other option (1/0.069 and 1/0.084). Again, future research is needed to determine the alternatives faced by these pet families, such as economic euthanasia, and whether they would have had other borrowing options, e.g., friends and family.

For the for-profit providers, the ability to meet existing clients in financial crisis offers financial and customer service opportunities. If we assume that those with a CSR of D, E, or F are not able to obtain other TPC financing, the data show that the percentage of total dollars of service that is not repaid is 5.6%. The G-rated accounts, the lowest possible rating, bring this collective default rate up to 6.9%. We note that some access to care models ask for a 20% discount from providers to targeted populations. A practice is financially better off self-insuring against the default at 6.0 or 6.9% of total services than providing 10 or 20% discounts, notably for those where a PLL abates the financial barrier. As previously stated, discounts also do not solve the cash flow issue for pet owners as the remaining cost is still an out-of-pocket expense. Further research is recommended to explore the levels at which providing payment options impacts clinics in terms of economic euthanasia decisions, client and staff stress, value perception and compliance with suggested care, outcomes for patients, and clinic revenue.

For the non-profit provider of veterinary services that procures donor dollars to subsidize part or all of the cost of providing veterinary care, PLL options offer a money multiplier effect to donor dollars, especially for the non-profit that does not offer any middle point on the spectrum between full price and full subsidy. If a donor has offered $100,000 to provide accessible sick or emergency veterinary care and those funds are used to cover the potential default of those with D, E, F, or G CSR codes, as a proxy for the inability to access other funding options to abate cash flow barriers, then the non-profit could potentially provide $1,449,275 of care to clients and their pets, nearly 15 times the number of family units (100,000/0.069), all while at a standard affordable price in their community vs. providing free care worth $100,000. Even if the non-profit was to work with the for-profit clinic and underwrite the G-rated CSR accounts alone, the lowest rating, 5.7 times the veterinary care could be provided over directly paying the bills for these pet families. Further research is recommended to identify how deeply PLL options can penetrate the population most challenged with any level of payment over time.

It is interesting that more than eight in ten payment plans are devised by practices without the use of the soft credit feature and CSR scoring. Further, the default rate among those without the CSR score is 6.2% of total service cost while the rate of those with CSR scoring is 4.9%. The majority of scores, where available, are in the range of likely declines for traditional credit suggesting that PLLs and non-credit-based payment options are a viable alternative to credit-based solutions for many clinics and clients. There will remain those that are unable to provide any payment for pet care. By more thoroughly reaching those between that level and people who are eligible for traditional funding, we will allocate scarce funds—such as discount and subsidy dollars—optimally and enable a great deal of veterinary care to those who need it.

Funders of access to care initiatives should look for providers to utilize payment options that address the number of pet families that are not eligible for traditional credit financing. Given the small number of non-profits in this analyzed dataset, it would appear that these clinics are lagging in the use of business tools. Additionally, accounts with non-profit clinics have higher down payment percentages, shorter terms, and lower financed amounts. By decreasing down payment percentages and increasing terms and financed amounts, non-profit clinics could support additional pet families who may require more flexibility in cash flow.

### Limitations and areas for further research

The researchers acknowledge several limitations to this analysis and opportunities for additional research. First, these data do not show whether individuals were declined for TCP options and what they would do if PLL payment plans were not an option, including the alternatives they and their pet would face such as economic euthanasia. This is an important area for further research in combination with collecting pet household characteristics and socio-economic data to extrapolate the gap in financial barriers that may be bridged by more payment options in veterinary medicine. Additionally, qualitative and quantitative research that identifies all the payment methods organizations provide and why individuals choose a payment plan over other options, and how payment options may influence decisions to treat are needed to provide clarity into the use of payment options for increasing access to care. Finally, client organizations do not indicate why CSR scores were provided to some accounts and not others. Additional qualitative research on the organization operations would give insight into how payment options are offered and used in practices and how practices communicate options with clients.

## Data availability statement

The data analyzed in this study is subject to the following licenses/restrictions: The dataset has been made available to Frontiers editors. Other researchers should contact the authors and VetBilling for permission to access data. Requests to access these datasets should be directed to HC, heather@opendoorconsults.org.

## Author contributions

SH and HC equally contributed to the analysis of the data and writing of the findings. All authors contributed to the article and approved the submitted version.

## Funding

The authors declare that this study received funding by a grant from Life of Riley at Spring Point Partners, a social impact organization based in Philadelphia, Pennsylvania. The funder was not involved in the study design, collection, analysis, interpretation of data, the writing of this article or the decision to submit it for publication.

## Conflict of interest

The authors declare that the research was conducted in the absence of any commercial or financial relationships that could be construed as a potential conflict of interest.

## Publisher's note

All claims expressed in this article are solely those of the authors and do not necessarily represent those of their affiliated organizations, or those of the publisher, the editors and the reviewers. Any product that may be evaluated in this article, or claim that may be made by its manufacturer, is not guaranteed or endorsed by the publisher.
