# Supplementary material for: Payment options: An analysis of 6 years of payment plan data and potential implications for for-profit clinics, non-profit veterinary providers, and funders to access to care initiatives
Source: Front Vet Sci. 2022 Aug 1;9:895532. doi: 10.3389/fvets.2022.895532 (PMC9376616; doi:10.3389/fvets.2022.895532)
Supplement: Supplementary file 1 [file Data_Sheet_1.docx]

***Supplementary Material***

# Supplementary Tables

**Table S1: % of Clients by Org Type and Year**

|  | **% of Clients by Org Type** | | | | |
| --- | --- | --- | --- | --- | --- |
| **Year** | **Emergency** | **Nonprofit** | **Small animal** | **Other** | **Grand Total** |
| **2016** | 9.3% | 1.6% | 86.8% | 2.3% | 100.0% |
| **2017** | 10.0% | 1.6% | 85.8% | 2.6% | 100.0% |
| **2018** | 9.0% | 1.4% | 86.5% | 3.2% | 100.0% |
| **2019** | 9.5% | 2.1% | 86.4% | 2.1% | 100.0% |
| **2020** | 12.1% | 2.9% | 81.6% | 3.3% | 100.0% |
| **2021** | 10.0% | 6.8% | 80.0% | 3.2% | 100.0% |
| **Grand Total** | 9.8% | 3.5% | 83.6% | 3.0% | 100.0% |

**Table S2: % of Accounts by Org Type and Year**

|  | **% of Accounts by Org Type** | | | | |
| --- | --- | --- | --- | --- | --- |
| **Year** | **Emergency** | **Nonprofit** | **Small animal** | **Other** | **Grand Total** |
| **2016** | 17.3% | 2.5% | 79.6% | 0.6% | 100.0% |
| **2017** | 19.7% | 7.5% | 71.5% | 1.3% | 100.0% |
| **2018** | 14.5% | 18.3% | 65.6% | 1.5% | 100.0% |
| **2019** | 19.3% | 18.8% | 60.5% | 1.4% | 100.0% |
| **2020** | 22.2% | 16.7% | 60.0% | 1.1% | 100.0% |
| **2021** | 27.5% | 15.9% | 55.7% | 0.9% | 100.0% |
| **Grand Total** | 21.2% | 15.4% | 62.3% | 1.2% | 100.0% |

**Table S3: % of Total Cost of Services by Org Type and Year**

|  | **% of Total Cost of Services by Org Type** | | | | |
| --- | --- | --- | --- | --- | --- |
| **Year** | **Emergency** | **Nonprofit** | **Small animal** | **Other** | **Grand Total** |
| **2016** | 29.1% | 0.8% | 69.2% | 0.9% | 100.0% |
| **2017** | 25.7% | 4.2% | 67.5% | 2.6% | 100.0% |
| **2018** | 24.2% | 11.9% | 61.0% | 2.8% | 100.0% |
| **2019** | 27.7% | 13.7% | 56.6% | 1.9% | 100.0% |
| **2020** | 32.5% | 12.1% | 54.5% | 0.9% | 100.0% |
| **2021** | 39.6% | 11.8% | 47.9% | 0.6% | 100.0% |
| **Grand Total** | 32.0% | 10.9% | 55.7% | 1.4% | 100.0% |

**Table S4: % of Total Financed Amounts by Org Type and Year**

|  | **% of Total Financed Amounts** | | | | |
| --- | --- | --- | --- | --- | --- |
| **Year** | **Emergency** | **Nonprofit** | **Small animal** | **Other** | **Grand Total** |
| **2016** | 25.7% | 1.1% | 72.2% | 1.0% | 100.0% |
| **2017** | 22.8% | 3.4% | 70.6% | 3.2% | 100.0% |
| **2018** | 21.1% | 9.2% | 66.4% | 3.3% | 100.0% |
| **2019** | 24.1% | 10.2% | 63.8% | 1.9% | 100.0% |
| **2020** | 30.6% | 8.4% | 60.1% | 0.9% | 100.0% |
| **2021** | 38.9% | 8.4% | 52.1% | 0.7% | 100.0% |
| **Grand Total** | 29.9% | 8.0% | 60.7% | 1.5% | 100.0% |
